# Methamphetamine Use and Methicillin-Resistant *Staphylococcus aureus* Skin Infections

**DOI:** 10.3201/eid1311.070148

**Published:** 2007-11

**Authors:** Adam L. Cohen, Carrie Shuler, Sigrid McAllister, Gregory E. Fosheim, Michael G. Brown, Debra Abercrombie, Karen Anderson, Linda K. McDougal, Cherie Drenzek, Katie Arnold, Daniel Jernigan, Rachel Gorwitz

**Affiliations:** * Centers for Disease Control and Prevention, Atlanta, Georgia, USA; † Georgia Division of Public Health, Atlanta, Georgia, USA; ‡ Kennestone Hospital, Marietta, Georgia, USA; § Northwest Georgia Health District 1–1, Rome, Georgia, USA

**Keywords:** Staphylococcus aureus, methicillin resistance, drug resistance, methamphetamine, street drugs, rural health, skin and soft tissue infections, research

## Abstract

Methicillin-resistant *Staphylococcus aureus* (MRSA) infections and methamphetamine use are emerging public health problems. We conducted a case–control investigation to determine risk factors for MRSA skin and soft tissue infections (SSTIs) in residents of a largely rural southeastern community in the United States. Case-patients were persons >12 years old who had culturable SSTIs; controls had no SSTIs. Of 119 SSTIs identified, 81 (68.1%) were caused by MRSA. Methamphetamine use was reported in 9.9% of case-patients and 1.8% of controls. After we adjusted for age, sex, and race, patients with MRSA SSTIs were more likely than controls to have recently used methamphetamine (odds ratio 5.10, 95% confidence interval 1.55–16.79). MRSA caused most SSTIs in this population. Transmission of MRSA may be occurring among methamphetamine users in this community.

## Abstract 2

Drug use may be contributing to the spread of MRSA in a rural southeastern US community.

Methicillin-resistant *Staphylococcus aureus* (MRSA) is a growing public health problem for urban and rural communities in the United States (*1**,**2*). Skin and soft tissue are the most common sites of MRSA infection, comprising >75% of MRSA disease (*3**,**4*). Skin and soft tissue infections (SSTIs), commonly caused by *S. aureus*, annually account for an estimated 11.6 million visits to hospital outpatient departments and emergency departments in the United States (*5*), and the percentage of SSTIs caused by MRSA in urban emergency departments increased from 29% in 2001 and 2002 to 64% in 2003 and 2004 (*6*). Some of the first reports of MRSA were in injection drug users in urban Detroit during the early 1980s (*7*,*8*).

Illegal methamphetamine use in the United States led to a rising number of methamphetamine-related hospital admissions from the early 1980s through the early 2000s (*9*). In 2004, 0.2% of the national population >12 years of age reported using methamphetamine in the previous month; 0.6% reported using it in the previous year (*10*). The prevalence of methamphetamine use has been reported to be >5% in at-risk populations such as young men from low-income, urban neighborhoods (*11*) and urban HIV-positive men who have sex with men (*12*).

On August 2, 2005, the Georgia Division of Public Health invited the Centers for Disease Control and Prevention (CDC) to assist in an on-site investigation of increased SSTIs among patients of a low-cost, fee-for-service clinic in rural Georgia. The clinic’s nurse practitioner had noted a history of methamphetamine use in multiple patients with SSTIs. Methamphetamine use has been associated with MRSA skin infections among urban HIV-positive men who have sex with men (*12*), but no study has evaluated the association of methamphetamine use and MRSA infection in a community with a large rural population. The objectives of this investigation were to define the public health effects and to determine risk factors, including methamphetamine use, for MRSA SSTI among residents of a community in the southeastern United States.

## Methods

### Epidemiologic Investigation

We conducted a prospectively enrolled case–control investigation at 3 emergency departments and 3 urgent care clinics in Georgia from September 6 through October 31, 2005. Two low-cost urgent care clinics that serve primarily low-income populations and all emergency departments in a 3-county area were included in an attempt to capture sites where methamphetamine users might seek medical care for SSTI. The third urgent care clinic was affiliated with one of the participating hospitals but was located in a neighboring county. According to the 2000 US Census, 43.9% of the population of these 3 counties lives in rural areas (*13*).

We defined a case-patient as a person >12 years of age with a laboratory culture–confirmed SSTI who came to a participating emergency department or clinic for treatment during the investigation period. Clinicians at participating institutions identified patients with culturable SSTIs and were asked to incise, drain, and culture all infected skin and soft tissue. Patients with SSTIs that were not culturable, such as simple cellulitis, were not included. Patients whose primary language was not English were enrolled if they could speak English fluently enough to answer survey questions. Patients with new or recurrent SSTI could also be enrolled; however, we excluded patients who had previously enrolled in the investigation.

Controls were patients >12 years of age with no current skin infection who were frequency matched by investigation site at a rate of 3 controls to 1 case-patient with MRSA infection. Controls were excluded if they reported a current skin infection or if infection was identified on physical examination. Persons could be enrolled as control patients if illness was minor and comparable in severity to an SSTI. For example, patients with major trauma and critically ill patients were excluded from control selection.

Upon seeking treatment, patients voluntarily consented to be interviewed by trained staff of the participating healthcare facilities, local public health departments, or CDC to identify SSTI case-patients. To ensure as much privacy as possible, the interviews were usually conducted in the patient’s room with no family or friends present. The interview survey contained questions about demographics, clinical history, and potential risk factors for SSTI. Each patient was asked a specific question about methamphetamine use: “In the past 3 months, have you used methamphetamine (crystal meth or meth)?” If the patient answered yes, 2 follow-up questions were asked: 1) “How did you take methamphetamine?” with the choices “smoked or inhaled,” “injected,” or “swallowed or took pills,” and 2) “Have you shared drug equipment or rinse water with anyone else, including a significant other?” To identify healthcare exposure, patients were asked whether they had had surgery or dialysis or if they had stayed overnight in a hospital within the previous 3 months. All patients, and their parents if the patients were <18 years of age, were given a letter explaining the investigation and asked to give verbal informed consent to enroll in the investigation. This investigation was deemed exempt from review by the CDC Institutional Review Board because it was part of a public health response by CDC and the Georgia Division of Public Health. We examined trends in *S. aureus* skin infections and cultures at one of the main emergency departments in our investigation by reviewing billing codes and laboratory microbiology reports from January 2004 through September 2005, the start of the case–control survey investigation.

### Laboratory Investigation

Specimens were obtained from at least 1 infection site in all case-patients. Staff at all 3 hospital emergency departments and the urgent care clinic affiliated with the respective hospital collected cultures and performed antimicrobial drug susceptibility testing at their facility. Two low-cost, urgent care clinics sent all cultures to CDC for culture and antimicrobial drug susceptibility testing. All 6 investigation sites sent both MRSA and methicillin-susceptible *S. aureus* (MSSA) isolates to CDC for further characterization.

All available isolates from methamphetamine users and a random sample of isolates not related to methamphetamine use from each of the 6 investigation sites were tested at CDC for antimicrobial susceptibility by the Clinical and Laboratory Standards Institute broth microdilution method (*14*). We tested for susceptibility to chloramphenicol, clindamycin, daptomycin, doxycycline, erythromycin, gentamicin, levofloxacin, linezolid, oxacillin, penicillin, rifampin, tetracycline, trimethoprim-sulfamethoxazole, and vancomycin. In addition, we performed the cefoxitin disk diffusion test to predict *mec*A-mediated resistance to oxacillin (*14*) and the D-zone test for inducible clindamycin resistance (*15*). Isolates were also tested by using PCR for genes encoding the staphylococcal cassette chromosome *mec* (SCC*mec*) resistance complex, Panton-Valentine leukocidin (PVL) cytotoxin, and toxic shock syndrome toxin (*16*). Chromosomal DNA was analyzed by pulsed-field gel electrophoresis (PFGE) after digestion with *Sma*I restriction endonuclease (*17*). The relatedness of PFGE patterns in different isolates was defined by using Dice coefficients and 80% relatedness by the unweighted pair-group method with arithmetic averages (Applied Maths, BioNumerics, Austin, TX, USA) (*18*).

### Statistical Methods

We conducted univariate analysis of the data to describe patient demographics and compared binary and categorical variables with the χ^2^ test; continuous variables were compared by using the *t* test with unequal variances. We evaluated risk factors for MRSA SSTIs by using conditional logistic regression with stratification by investigation site. Risk estimates were adjusted for age (categorized as <18 years, 19–34 years, 35–64 years, and >65 years), sex, and race (categorized as white and nonwhite) because they were potential confounding variables.

## Results

### Epidemiologic Investigation

We identified 119 case-patients with skin infections in the investigation. MRSA was isolated from 81 (68.1%) of the skin and soft tissue cultures, MSSA from 20 (16.8%), and bacteria other than *S. aureus* from 18 (15.1%) (Table 1). Compared with controls with no skin infection, a higher percentage of patients with MRSA SSTIs were male (p<0.001). The proportion of patients that were male did not differ significantly between controls and patients with either MSSA or non–*S. aureus* SSTIs (p = 0.67 for MSSA, p = 0.12 for non–*S. aureus*) or between patients with MRSA and MSSA SSTIs (p = 0.16).

**Table 1:** Demographic characteristics of study participants with (case-patients) and without (controls) skin and soft tissue infections (SSTIs)*

Characteristic	Patients with SSTIs			Patients without SSTIs (N = 284), no. (%)
	MRSA (N = 81), no. (%)	MSSA (N = 20), no. (%)	Other† (N = 18), no. (%)	
Age, y				
<18	12 (14.8)	0	2 (11.1)	18 (6.3)
19–34	30 (37.0)	13 (65.0)	8 (44.4)	102 (35.9)
35–64	35 (43.2)	6 (30.0)	7 (38.9)	135 (47.5)
>65	4 (4.9)	1 (5.0)	1 (5.6)	29 (10.2)
Male sex‡	48 (59.3)§	8 (40.0)	10 (55.6)	104 (36.6)
Race¶				
White	73 (90.1)	18 (90.0)	16 (88.9)	244 (85.9)
Black	5 (6.2)	2 (10.0)	2 (11.1)	36 (12.7)
Other	3 (3.7)	0	0	3 (1.1)
Hispanic ethnicity#	2 (2.5)	0	0	4 (1.4)

*MRSA, methicillin-resistant *Staphylococcus aureus*; MSSA, methicillin-susceptible *S. aureus*. †Bacteria other than *S. aureus* isolated from SSTI in our investigation included other *Staphylococcus* spp., *viridans group streptococci*, Group B *Streptococcus*, *Enterobacter cloacae*, *Stenotrophomonas maltophilia*, and mixed flora. ‡6 records did not indicate sex (1 MRSA case, 1 MSSA case, and 4 controls). §p<0.0001, when compared with controls. ¶For 1 control, race was not indicated. #3 records did not indicate ethnicity (2 MRSA cases, 1 other skin infection).

Fifteen patients who reported recently using methamphetamine were identified: 8 with MRSA SSTIs, 2 with MSSA SSTIs, and 5 controls. Half (8 [53.3%]) of the methamphetamine users were male. Ten percent of patients with MRSA skin infections (8/81) reported using methamphetamine in the past 3 months, significantly more than the 2% of controls (5/283) who reported this behavior (p<0.001). After adjusting for age, sex, and race, we determined that patients with MRSA SSTI were significantly more likely to have recently used methamphetamine than were controls (adjusted odds ratio [AOR] 5.10, 95% confidence interval [CI] 1.55–16.79) (Table 2). Of the 8 methamphetamine users with MRSA SSTIs, most (5 [62.5%]) smoked or inhaled the drug. Only 1 (12.5%) injected the drug, and 1 (12.5%) took the drug orally. For 1 methamphetamine user with MRSA SSTI, we could not determine the route of drug administration. Of the 8 methamphetamine users with MRSA SSTIs in our investigation, 2 (25.0%) reported sharing drug equipment or rinse water with other persons; we did not have information on drug-sharing behavior for 1 methamphetamine user with a MRSA SSTI.

**Table 2:** Risk factors for MRSA skin and soft tissue infection*

Risk factors	Case-patients, no. (%)	Controls, no. (%)	Crude OR (95% CI)	Adjusted OR† (95% CI)
Drug use and medical history				
Recent skin infection‡	34 (42.0)	22 (7.8)	8.41 (4.54–15.59)	7.92 (4.10–15.28)
Recent methamphetamine use‡	8 (9.9)	5 (1.8)	5.64 (1.80–17.69)	5.10 (1.55–16.79)
Antimicrobial agents within 6 months	40 (49.4)	114 (40.1)	1.43 (0.87–2.34)	1.52 (0.89–2.60)
Recent hospitalization, surgery, or dialysis‡	8 (9.9)	27 (9.5)	1.06 (0.46–2.44)	1.24 (0.51–2.97)
Diabetes	10 (12.4)	23 (8.1)	1.61 (0.73–3.57)	2.03 (0.83–4.98)
Liver disease	1 (1.2)	9 (3.2)	0.38 (0.05–3.07)	0.59 (0.70–4.91)
Contact exposure				
Household contact with someone with skin infection	21 (25.9)	27 (9.5)	3.26 (1.72–6.17)	3.19 (1.58–6.48)
Crowding (>1 person/bedroom)	44 (54.3)	111 (39.1)	2.06 (1.22–3.45)	1.78 (1.004–3.15)§
Recent sexual contact‡	48 (59.3)	182 (64.1)	0.85 (0.51–1.42)	0.68 (0.38–1.22)
Recent sexual contact with someone with skin infection‡	7 (8.6)	6 (2.1)	4.28 (1.40–13.08)	5.42 (1.68–17.50)
Recent contact sports‡	9 (11.1)	11 (3.9)	2.92 (1.17–7.31)	1.37 (0.47–4.03)
Recent jail‡	4 (4.9)	9 (3.2)	1.46 (0.44–4.90)	1.75 (0.48–6.42)
Hygiene practices				
Frequent skin picking	17 (20.1)	24 (8.5)	2.77 (1.40–5.47)	2.53 (1.22–5.23)
Bathe less than daily	5 (6.2)	31 (10.9)	0.50 (0.19–1.34)	0.56 (0.19–1.67)

*MRSA, methicillin-resistant *Staphylococcus aureus*; OR, odds ratio; CI, confidence interval. †All models are adjusted for age, sex, race, and methamphetamine use, except the model for methamphetamine use, which is adjusted only for age, sex, and race. ‡Recent = within the 3 months prior to survey. §p = 0.048.

In our study population, having had a skin infection within the previous 3 months was the factor most strongly associated with current MRSA skin infection (AOR 7.92, 95% CI 4.10–15.28) (Table 2). Recent sexual contact with someone with a skin infection was also a significant risk factor for MRSA skin disease (AOR 5.42, 95% CI 1.68–17.50), when compared with recent sexual contact with a person without a skin infection. Frequent skin-picking behavior was independently associated with MRSA SSTI (AOR 2.53, 95% CI 1.22–5.23). Crowded living conditions, defined as >1 person per bedroom, had a small but significant association with MRSA SSTI (AOR 1.78, 95% CI 1.004–3.15).

Only 10% of MRSA case-patients had healthcare-associated risk factors traditionally associated with MRSA infection, namely, recent hospitalization, surgery, or dialysis. Additional factors not significantly associated with MRSA SSTI in our study population included use of antimicrobial agents in the previous 6 months, recent stays in a jail or prison, bathing less than daily, history of diabetes or liver disease, recent tattoo or body piercing, and participation in contact sports in the previous 3 months. In addition, very few or no patients were HIV positive (2 [0.5%]), homeless (0), or recently had sex with someone of the same sex (7 [1.6%]), suggesting that none of these were significant risk factors for MRSA SSTI in this population.

**Figure 1:**
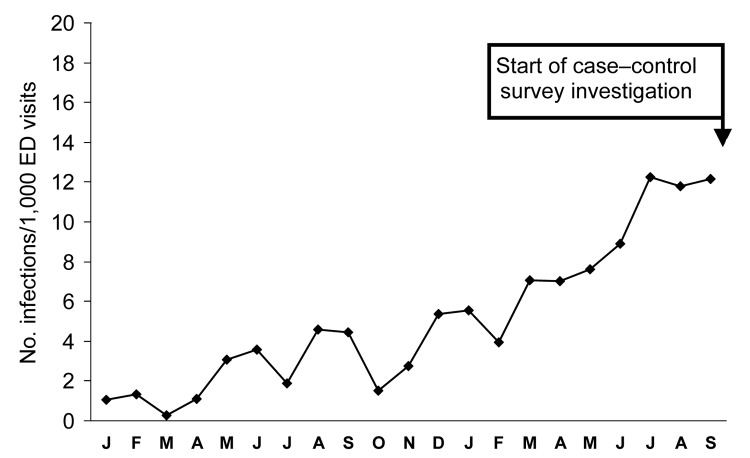
Number of *Staphylococcus aureus* skin infections at a southeastern United States emergency department, January 2004–September 2005.

The number of visits for *S. aureus* skin infections at one of the main emergency departments in our investigation increased from ≈1 per 1,000 emergency department visits to 12 per 1,000 visits over the 20 months leading up to the investigation (Figure 1). This emergency department accounted for 46.2% of all study participants in our investigation. Over the same period, MRSA infections increased from 2 to 38 per month in the same emergency department. Most emergency department *S. aureus* cultures for both SSTIs and non-SSTIs were resistant to methicillin, with the prevalence of methicillin-resistance remaining stable over the same 20-month period (median 82%, range 50–100%).

### Laboratory Investigation

MRSA (n = 32) and MSSA (n = 13) isolates tested were commonly susceptible to clindamycin, daptomycin, doxycycline, gentamicin, levofloxacin, linezolid, rifampin, tetracycline, trimethoprim-sulfamethoxazole, and vancomycin (Table 3). None of the MRSA isolates and only 1 (7.7%) of the MSSA isolates had inducible clindamycin resistance. MRSA susceptibility patterns of isolates from methamphetamine users and nonusers were similar, except that both MRSA isolates susceptible to erythromycin were found in those who did not use methamphetamine. The MSSA isolate from a methamphetamine user was susceptible to all but penicillin.

**Table 3:** Antimicrobial susceptibility patterns and toxin gene presence of selected MRSA and MSSA isolates*

Antimicrobial agent or toxin	MRSA isolates† (N = 32), no. (%)	MSSA isolates (N = 13), no. (%)
Antimicrobial susceptibility		
Chloramphenicol	32 (100.0)	10 (76.9)‡
Clindamycin	32 (100.0)	12 (92.3)
Inducible resistance (D-zone test)	0	1 (7.7)
Daptomycin	32 (100.0)	13 (100.0)
Doxycycline	32 (100.0)	13 (100.0)
Erythromycin	2 (6.5)	6 (46.2)
Gentamicin	32 (100.0)	13 (100.0)
Levofloxacin	27 (84.4)	12 (92.3)
Linezolid	32 (100.0)	13 (100.0)
Penicillin	0	2 (15.4)
Rifampin	32 (100.0)	13 (100.0)
Trimethoprim-sulfamethoxazole	32 (100.0)	13 (100.0)
Vancomycin	32 (100.0)	13 (100.0)
Toxin gene presence		
Panton-Valentine leukocidin	32 (100.0)	5 (38.5)
TSST–1	0	0

*MRSA, methicillin-resistant *Staphylococcus aureus*; MSSA, methicillin-susceptible *Staphylococcus aureus*; TSST, toxic shock syndrome toxin. †Methicillin resistance was determined by the oxacillin MIC and disk diffusion using a 30-μg cefoxitin disk (*14*). ‡Three (23.1%) isolates had intermediate resistance to chloramphenicol.

**Figure 2:**
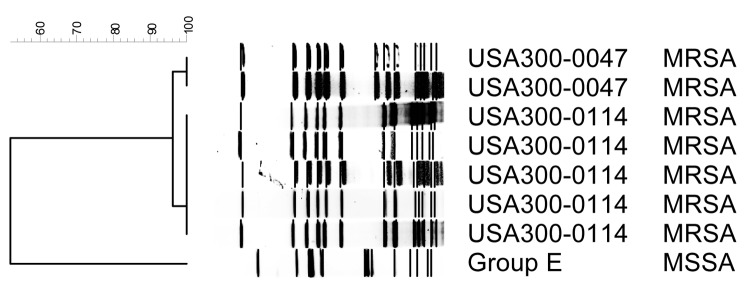
Dendrogram of pulsed-field types for methicillin-resistant *Staphylococcus aureus* (MRSA) and methicillin-susceptible *S. aureus* (MSSA) isolated from methamphetamine users.

We detected genes for PVL in all MRSA isolates and 5 (41.7%) MSSA isolates; however, the MSSA isolate from a methamphetamine user did not carry the PVL locus. All available MRSA isolates from 6 methamphetamine users and 21 nonusers of methamphetamine had type IV SCC*mec* resistance complex and were PFGE type USA300. Most of the MRSA isolates were a single strain, PFGE type USA300-0114 (4 [66.7%] were methamphetamine users, 15 [71.4%] were non-methamphetamine users) (Figure 2). One third (33.3%) of MRSA isolates from methamphetamine users and one fifth (19.0%) of MRSA isolates from non-methamphetamine users were variants of USA300-0114, such as USA300-0047.

## Discussion

MRSA caused over two thirds of all skin infections in the Georgia community we investigated, which is among the highest reported rates of MRSA in SSTI nationwide (*16*). We found that many previously known risk factors for MRSA skin infection, such as recent skin infection and household contact with someone with a skin infection (*19*), were common in this population. However, we also identified a novel association between MRSA skin infections and methamphetamine use in a community with a large rural population. Methamphetamine use was reported in nearly 1 in 10 patients with MRSA SSTI and was more common in patients with MRSA skin infections than in patients without skin infections. While most community-associated MRSA SSTI occur in persons without defined risk factors (*16*), some settings such as prisons and military training facilities appear to facilitate and amplify MRSA transmission (*20**,**21*). A similar amplification of transmission may be occurring among methamphetamine users in this community.

Methamphetamine use is associated with a number of socioeconomic and behavioral risk factors that may predispose persons to MRSA SSTI. We found that MRSA SSTI was associated with living with someone with a skin infection, which may increase skin contact with infected persons. Skin-picking was also associated with MRSA SSTI. Methamphetamine use causes formication, a sensation of something crawling on the body or under the skin, which can lead to skin-picking behavior, skin breakdown, and portals of infection. Other poor hygiene habits that can break the skin, such as fingernail biting, have been associated with MRSA SSTI (*12*). Methamphetamine use may be associated with limited access to medical care, stays in correctional facilities, and homelessness, all of which have been associated with MRSA SSTI in previous studies (*20**,**22*). However, our investigation did not find these to be significant risk factors for MRSA SSTI in this population.

Methamphetamine use has been associated with HIV (*23*) and sexually transmitted bacterial infections (*24*), purportedly from increased unprotected sex related to the sexually stimulating property of the drug. A study among urban HIV-positive men who have sex with men found that, in addition to methamphetamine use, use of other sexually stimulating drugs such as nitrates (“poppers”) and sildenafil (e.g., Viagra) was associated with MRSA SSTI (*12*). These previous findings and the results of the current investigation suggest that the use of methamphetamine and other sexually stimulating drugs may increase direct skin-to-skin sexual contact and transmission of MRSA, which can be transmitted through sexual contact (*25*). We found an increased risk for MRSA SSTI in case-patients who had recently had sex with someone with a skin infection.

Injection of the drug may act as a method of introducing the bacteria into the skin if users fail to clean injection sites or share drug paraphernalia and other potentially contaminated items (*26*). Injection of methamphetamine can lead to transmission of bloodborne pathogens when injection equipment is shared, as demonstrated in an outbreak of hepatitis B among methamphetamine users in Wyoming (*27*). A recent case series of 14 patients with MRSA necrotizing fasciitis found that 43% of the patients had current or past injection drug use (*28*). In contrast to early reports of MRSA in urban injection drug users, our investigation suggested that MRSA skin infections in methamphetamine users are not necessarily due to unclean drug injection. Few methamphetamine users in our population injected the drug or shared equipment; rather, the methamphetamine users in this community commonly smoked or inhaled the drug.

The absolute number of SSTIs at 1 emergency department in this investigation increased during the 18 months preceding the investigation, but the percentage of MRSA isolates was stable over that period. This increase in SSTIs led to a concomitant increase in MRSA SSTIs, which were more common among men, and echoes repeated reports of MRSA SSTI outbreaks in male populations (*20**,**29*). This sex difference was not due to increased methamphetamine use in men in our population, since our population of surveyed methamphetamine users was evenly divided between the sexes. We also did not find many MRSA infections in nonwhite patients. This finding contrasts with previous reports of higher incidence of MRSA SSTIs in African Americans in urban centers compared to other races (*4*) and likely reflects the predominantly white racial distribution (98.9%) in these 3 rural southeastern US counties (*13*).

The laboratory investigation found that the most common MRSA strain causing community SSTI was PFGE type USA300-0114, a highly conserved strain that has been implicated in multiple community outbreaks (*19*). The second most common MRSA strain in this community, and the only other strain found among methamphetamine users, was USA300-0047, which has only a 1-band difference from USA300-0114. MRSA SSTIs in methamphetamine users were not due to a novel or unusual strain of MRSA but rather the most common strain of MRSA in community settings across the United States.

Our investigation is subject to some limitations. First, we did not identify nor do we have data on every SSTI patient who came to the participating clinics and emergency departments for treatment; not every patient with SSTI provided specimens for culture or participated in our survey. Second, we relied on patient report of methamphetamine use and did not conduct drug screens for confirmation. Third, we excluded patients who could only speak Spanish, which may have added to the low number of Hispanic study participants and affected the generalizability of the results. However, Hispanic, foreign-born, and non-English primary speakers each comprise only 5%–10% of the population of these 3 counties (*13*). Fourth, we were unable to test for other physiologic theories of why methamphetamine use may be associated with MRSA, which include weakening the immune system and predisposing users to MRSA carriage by changing the nasal environment. Fifth, we were unable to test whether methamphetamine itself or drug paraphernalia were contaminated with MRSA. Lastly, transmission of MRSA in this population may have occurred in either the community or in the healthcare setting; for some cases, we were unable to determine the origin of the community strains.

Our investigation has direct implications for clinicians. Most clinicians in the participating emergency departments and urgent care clinics did not routinely drain or culture SSTIs. Incision and drainage is a primary therapy for SSTI, and empiric antimicrobial drug therapy may be given in addition to incision and drainage (*30*). Because of the growing and changing resistance patterns in the community, clinicians should consider culturing SSTI (*30*). In this population, antimicrobial agents currently recommended for treatment of MRSA (e.g., clindamycin, doxycycline, and trimethoprim-sulfamethoxazole) would be appropriate choices for empiric treatment of outpatient SSTI because of low prevalence of resistance (*30*.) Patients should also be educated about the risks for transmission through household and sexual skin-to-skin contact. Transmission of MRSA in this community is likely due to various factors, and some of these community strains may have been transmitted through healthcare exposure.

Patients with MRSA SSTIs who seek treatment may help clinicians identify a vulnerable population of methamphetamine users. Prevention measures, such as improved hygiene and correct care for wounds, may be helpful when directed at methamphetamine users. However, MRSA SSTIs in methamphetamine users may also impact family and community members who do not use methamphetamine. The same strains of MRSA were circulating among both users and nonusers in our investigation. Public health officials and clinicians should be aware of proper identification, appropriate treatment, prevention of MRSA SSTIs, and the link between methamphetamine use and these SSTIs.
